# Core transcriptome network modulates temperature (heat and cold) and osmotic (drought, salinity, and waterlogging) stress responses in oil palm

**DOI:** 10.3389/fpls.2024.1497017

**Published:** 2024-12-23

**Authors:** Fong Chin Lee, Wan-Chin Yeap, Shao Yong Kee, Harikrishna Kulaveerasingam, David Ross Appleton

**Affiliations:** ^1^ Biotech and Breeding, SD Guthrie Technology Centre Sdn. Bhd., Serdang, Selangor Darul Ehsan, Malaysia; ^2^ SD Guthrie Research Sdn. Bhd., Banting, Selangor Darul Ehsan, Malaysia

**Keywords:** oil palm, abiotic stresses, core transcriptome, RNA-seq, differential expressed genes

## Abstract

Oil palm (*Elaeis guineensis*) yield is impacted by abiotic stresses, leading to significant economic losses. To understand the core abiotic stress transcriptome (CAST) of oil palm, we performed RNA-Seq analyses of oil palm leaves subjected to drought, salinity, waterlogging, heat, and cold stresses. A total of 19,834 differentially expressed genes (DEGs) were identified. Cold treatment induced the highest number of DEGs (5,300), followed by heat (4,114), drought (3,751), waterlogging (3,573), and, lastly, salinity (3096) stress. Subsequent analysis revealed the CAST of oil palm, comprising 588 DEGs commonly expressed under drought, salinity, waterlogging, heat, and cold stress conditions. Function annotation of these DEGs suggests their roles in signal transduction, transcription regulation, and abiotic stress responses including synthesis of osmolytes, secondary metabolites, and molecular chaperones. Moreover, we identified core DEGs encoding kinases, ERF, NAC TFs, heat shock proteins, E3 ubiquitin-protein ligase, terpineol synthase, and cytochrome P450. These core DEGs may be potential key modulators that interplay in triggering rapid abiotic stress responses to achieve delicate equilibrium between productivity and adaptation to abiotic stresses. This comprehensive study provides insights into the key modulators in the CAST of oil palm, and their potential applications as markers for selecting climate-resilient oil palms or opportunities to develop future climate resilient oil palm using genome editing.

## 1 Introduction

Rapid climate change has caused unfavorable environmental effects including drought, salinity, heat, waterlogging, and cold, collectively known as abiotic stress. These abiotic stresses affect plant growth and development, preventing plants from reaching their full potential and causing plant death if the stress level exceeds their tolerance. These adverse effects led to a drastic decrease in productivity and quality and substantial economic losses in various crops including maize and soybean, which are severely affected by drought (Zipper et al., 2016), while salinity stress negatively affects the rice grain filling, leading to yield decrease (Li et al., 2023). If crops fail to respond and adapt fast to abiotic stresses caused by climate change, poor crop productivity will eventually threaten global food security (Warsame et al., 2023).

Plants are constantly exposed to abiotic stresses from the external environment. To circumvent the negative impact from abiotic stress, plants have adopted different stress-response strategies including stress avoidance, escape, and tolerance mechanisms, modulating the morphological, biochemical, and physiological changes at the cellular and molecular level (Kashyap et al., 2021; Saharan et al., 2022). These short-term and long-term responses determined by the severity of abiotic stress and the physiological stage of the plants lead to plant survival and to achieve a delicate equilibrium between productivity and adaptation (Shavrukov et al., 2017; Ali et al., 2022).

Achieving a balance between energy consumption and generation increases the chances of plant survival in harsh environments (Suzuki et al., 2012). To achieve that, plants must be able to perceive the external and internal signals fast and concisely. Different signaling pathways are involved in plant abiotic stress responses including abscisic acid (ABA)-dependent or -independent, mitogen-activated protein kinase (MAPK), and calcium cascade transduction (Xiang et al., 2007; Kong et al., 2011; Bharti et al., 2021). During unfavorable conditions, a stress signal is first perceived by a plant organ and cascaded to different parts of plant cells through signaling molecules such as plant hormones that include ABA, auxin and ethylene, secondary messengers like reactive oxygen species (ROS), calcium ion (Ca^2+^), nitric oxide (NO), and secondary metabolites (Boncan et al., 2020; Zhu et al., 2021; Foyer and Hanke, 2022; Waadt et al., 2022). A signaling cascade transduction and stress-responsive transcription factors (TFs) in the nucleus stimulate and reprogram the transcription of stress-responsive genes to produce functional proteins involved in metabolic processes including photosynthesis, respiration, glycolysis, and lipid metabolism. Those TFs implicated in abiotic stress response include WRKY (Li et al., 2020), APETALA2/ethylene responsive factor (AP2/ERF) family (Wu et al., 2022), MYB (Wang et al., 2021), and basic helix-loop-helix (bHLH) (Qian et al., 2021). Precise reprogramming of abiotic stress-responsive gene expression will contribute to plant resistance and tolerance to abiotic stresses.

Numerous reports highlighted the close relationship between ROS signaling, redox homeostasis, and photosynthesis in abiotic stress responses (Sachdev et al., 2021). Under unfavorable conditions, photosynthesis is negatively impacted, leading to stomata closure and reduction in carbon dioxide level within cells (Sharma et al., 2020). The ROS scavenging enzymes are then produced to regulate the cellular ROS concentration, to protect plant cells from oxidative damage that is detrimental to plant health. These ROS scavenging enzymes include superoxide dismutase (SOD), ascorbate peroxidase (APX), catalase (CAT), glutathione reductase (GR), glutathione S-transferase (GST), and peroxiredoxin (PRX) (You and Chan, 2015). Meanwhile, osmo-protectants are synthesized to adjust cellular osmotic pressure including amino acids (proline), sugars (trehalose and sucrose), and alcohols (Mishra et al., 2022). Homeostasis of cellular proteins is maintained by chaperones such as heat shock proteins (HSPs) and small heat shock proteins (sHSPs) that safeguard proteins against degradation induced by abiotic stresses (Kummari et al., 2020; Neto et al., 2020). These responses enable plants to achieve an ideal homeostatic state for adapted growth and development under harsh environments.

Oil palm (*Elaeis guineensis*) is a highly productive oil crop, contributing to approximately 40% of the global vegetable oil demand and ranked as the most consumed vegetable oil globally (Murphy et al., 2021; John Martin et al., 2022). Constant exposure to various abiotic stresses has negatively impacted oil palm yield performance (Abubakar et al., 2022). The severe El Niño events in 2015–2016 resulted in abnormal frond development and low floral sex ratio that negatively impacted palm oil yield (Kamil and Omar, 2016, 2017). The increase of air temperature due to the El Niño event will impose severe water stress on oil palm and was predicted to cause a decrease in total annual oil palm yield (Oettli et al., 2018). Waterlogging caused lower oil palm yield in high-flooded areas as compared with non-flooded areas (Fadila et al., 2024). Moreover, scarcity in arable land and increasing global population have amplified the effort to improve the current oil palm planting materials with climate resilience traits. To achieve this, a genome-wide gene expression analysis using an omics approach has been applied to understand the oil palm responses and tolerance to abiotic stress, including drought (Wang et al., 2020; Leão et al., 2022; Salgado et al., 2022), salinity (Bittencourt et al., 2022; Ferreira et al., 2022), waterlogging (Nuanlaong et al., 2020; Lim et al., 2023), heat (Maryanto et al., 2021), and cold (Saand et al., 2022). A multi-omics integration (MOI) study on oil palm in response to drought and salinity revealed similarities, particularly in the cysteine and methionine metabolisms (Leão et al., 2022). Transcriptome analysis of oil palm roots under waterlogging stress revealed the importance of ROS-scavenging enzymes in conferring waterlogging tolerance (Nuanlaong et al., 2020). These studies establish a foundation for oil palm responses to individual or dual abiotic stresses. However, no comprehensive study was carried out to unravel the oil palm core abiotic stress transcriptome (CAST) to all five abiotic stresses influenced by climate change, including drought, salinity, waterlogging, heat, and cold stresses. As such, we performed a comparative study of oil palm leaf transcriptome subjected to drought, salinity, waterlogging, heat, and cold stress to understand oil palm core transcriptomic response networks to multiple abiotic stresses. These findings will enhance the current knowledge in oil palm stress responses to multiple abiotic stresses. Moreover, the identification of core DEGs provides opportunities in developing climate-resilient oil palms capable of withstanding unpredictable climate changes through marker-selection breeding or genome editing tools.

## 2 Materials and methods

### 2.1 Plant materials, growth condition, and treatments

Six-month-old oil palm *Dura* (Deli *Dura*) seedlings obtained from SD Guthrie Research Sdn. Bhd., Malaysia were planted in polybags filled with equal amount of topsoil. They were acclimatized at 28°C under controlled illumination (350 μmol m^−2^ s^−1^), 12 h of light followed by 12 h of darkness each day in a greenhouse environment for 1 month prior to exposure to different types of abiotic stress treatments for a duration of 2 weeks. All treatments and control were conducted at 28°C except for cold treatment at 10°C and heat treatment at 40°C. Prior to the treatment initiation, all seedlings were well watered to maintain volumetric water content (vwc) at approximately 0.32. For control, heat and cold treatments, the watering schedule was maintained at 200 mL of tap water daily, whereas seedlings under drought treatment received no water for 2 weeks, resulting in vwc dropping to 0.13. For salinity treatment, seedlings were irrigated with 200 mL of 200 mM NaCl daily, and for waterlogging treatment, the water level was maintained at 1 inch above the soil level. Each stress treatment and control were conducted in six biological replicates, respectively. For transcriptome profiling, the third leaf of three oil palm seedlings per replicate were harvested from the three randomly selected biological replicates for each treatment and control. The leaf samples were flash-frozen in liquid nitrogen and stored at −80°C until RNA isolation.

### 2.2 RNA extraction, library preparation, and Illumina sequencing

Total RNA of oil palm leaves was extracted from control and treated seedlings using the MN Nucleospin RNA Plant Kit (Macherey-Nagel, Germany) according to the manufacturer’s instructions. The RNA contamination, purity, concentration and integrity were determined using agarose gel electrophoresis, a NanoPhotometer^®^ spectrophotometer (IMPLEN, CA, USA), the Qubit^®^ RNA Assay Kit of the Qubit^®^ 2.0 Fluorometer (Life Technologies, CA, USA), and an RNA Nano 6000 Assay Kit on the Bioanalyzer 2100 system (Agilent Technologies, CA, USA), respectively. Samples of high quality and quantity were subjected to library preparation. A total amount of 3 μg of RNA per sample was used for sequencing library generation using the NEBNext^®^ Ultra™ RNA Library Prep Kit from Illumina^®^ (NEB, USA) following the manufacturer’s recommendations. Before the library sequencing, each library concentration was quantified using a Qubit^®^ 2.0 Fluorometer (Life Technologies, CA, USA) and diluted to 1 ng/μL for insert size check using a Bioanalyzer 2100 system (Agilent Technologies, CA, USA). These libraries were sequenced in 150-nt paired-end mode using the HiSeq2000 platform (Illumina, San Diego, CA, USA) at Novogene, Beijing, China.

### 2.3 Sequencing reads mapping and quantification of gene expression

The raw data from Illumina HiSeq™ were transformed into sequenced reads by base calling. Raw data were recorded in a FASTQ file, containing the sequence information (reads) and the corresponding sequencing quality information. Reads containing adapter, poly-N (N >10%), and low-quality reads were removed. Those clean reads were then mapped to the oil palm reference genome accession number PRJNA192219 deposited in the NCBI (Singh et al., 2013), using TopHat2 v2.0.12 as the mapping tool (Kim et al., 2013). The mismatch parameter was set to 2 and other parameters were set to default. The HTSeq software was used to analyze gene expression levels by counting aligned reads mapped to genes (Anders et al., 2015). Each gene expression level was quantified using fragments per kilobase of transcript over million mapped reads (FPKM), which normalized the total sequencing depth and gene length for read counts at the same sequencing level (Kim et al., 2013). The FPKM value was set at 0.1 or 1 as the threshold for determining the gene expression. The raw transcriptome data of all 18 samples were deposited into the Sequence Read Archive (SRA) of NCBI under accession number PRJNA775831. The FPKM values of all these samples were deposited in the Gene Expression Omnibus (GEO) database of NCBI under accession number GSE14069613.

### 2.4 Differential gene expression, gene ontology, and KEGG enrichment analyses

Differential gene expression analysis was then performed using the DESeq R package (1.18.0) (Anders and Huber, 2012) based on the negative binomial distribution model. The resulting *p*-values were adjusted using the Benjamini and Hochberg (Benjamini and Hochberg, 1995) approach for controlling the false discovery rate (FDR). Genes with an adjusted *p*-value < 0.05 and |log_2_fold change (FC)| ≥1 were considered as differentially expressed genes (DEGs). The CAST of oil palm was constituted by the DEGs that were commonly identified in the transcriptome of all five abiotic stresses, regardless of their expression pattern. Gene ontology (GO) and Kyoto Encyclopedia of Genes and Genomes (KEGG) enrichment analyses were carried out using shinyGO (Ge et al., 2020). The GO enrichment analysis classified the DEG functions into three categories: cellular component, biological process, and molecular function. Categories and pathways with FDR *p*-value < 0.05 were considered as significantly enriched (Benjamini and Hochberg, 1995). The transcription factor analysis of DEGs was conducted using the iTAK program V1.2 according to default parameters (Zheng et al., 2016). Volcano plots were generated using the ggplot2 package in R. UpSet plot, bubble plot, and hierarchy clustering were constructed using the SRplot tool (Tang et al., 2023). Jvenn was used to plot the Venn diagram (Bardou et al., 2014).

### 2.5 Quantitative RT-PCR analysis

The first-strand cDNA was synthesized from total RNA of oil palm tissue using the Maxima First Strand cDNA Synthesis Kit (Thermo Scientific, USA) and quantified using StepOne Plus (Applied Biosystems, USA) and Fast SYBR Green Master Mix (Applied Biosystems, USA) according to the manufacturer’s instructions. Dissociation curves were generated to verify the amplification specificity. Independent quantitative RT-PCR (qRT-PCR) runs were conducted in both biological and technical triplicates for different abiotic stress treatments and the calibrated normalized relative quantity (CNRQ) values of transcripts were calculated using delta–delta Ct method (Livak and Schmittgen, 2001). Expression of target genes was normalized to *Gibberellin-responsive protein 2* (*EgGRAS*), *Cyclophilin 2* (*EgCyp2*), and *Pre-mRNA splicing factor SLU7* (*EgSLU7*) (Yeap et al., 2014).

## 3 Results

### 3.1 Transcriptomic profiles and DEGs of oil palm leaves in response to abiotic stresses

To enhance the understanding of oil palm transcriptional changes in response to multiple abiotic stresses, we performed RNA-Seq analysis in Deli *Dura* oil palm leaves. In this study, 18 RNA-Seq libraries (three biological replicates per treatment) were constructed for oil palm under control and treatments including drought, salinity, waterlogging, heat, and cold. Approximately 843 million clean reads and 126.46 Gb clean bases were generated from these 18 libraries with mapping rates to the oil palm reference genome, ranging from 73.26% to 82.41% (**Table 1**). Among these samples, heat-treated samples exhibited the highest mapping rate at more than 81% (**Table 1**).

**Table 1:** Summary of the sequence data and alignment statistics result for each sample.

Samples	Raw reads	Clean reads	Clean bases	Total mapped	Uniquely mapped	Mapping rate
Control_1	46346660	43826976	6.57G	34454485	34184128	78.00%
Control_2	49862810	47241832	7.09G	34860834	34607484	73.26%
Control_3	47402594	44932664	6.74G	35572563	35333391	78.64%
Drought_1	54773672	52262722	7.84G	42400131	42064309	80.49%
Drought_2	43007412	41318284	6.20G	32892954	32675508	79.08%
Drought_3	45428296	43763410	6.56G	35212964	34986700	79.95%
Salinity_1	49294556	46763496	7.01G	37206278	37004959	79.13%
Salinity_2	53431790	50876600	7.63G	41410756	41085872	80.76%
Salinity_3	54283762	51631836	7.74G	40807468	40451408	78.35%
Waterlogging_1	47946264	46113210	6.92G	36754635	36527243	79.21%
Waterlogging_2	48435170	46636170	7.00G	36997110	36770124	78.84%
Waterlogging_3	37450098	36012808	5.40G	28475516	28273001	78.51%
Heat_1	51508760	49749990	7.46G	40711543	40410634	81.23%
Heat_2	53744834	51906452	7.79G	43039645	42776187	82.41%
Heat_3	50084774	48388806	7.26G	40057423	39832383	82.32%
Cold_1	50524182	48791952	7.32G	39408890	39127109	80.19%
Cold_2	46794014	45115870	6.77G	36500149	36305800	80.47%
Cold_3	49575890	47808808	7.17G	38957044	38686761	80.92%

**Figure 1:**
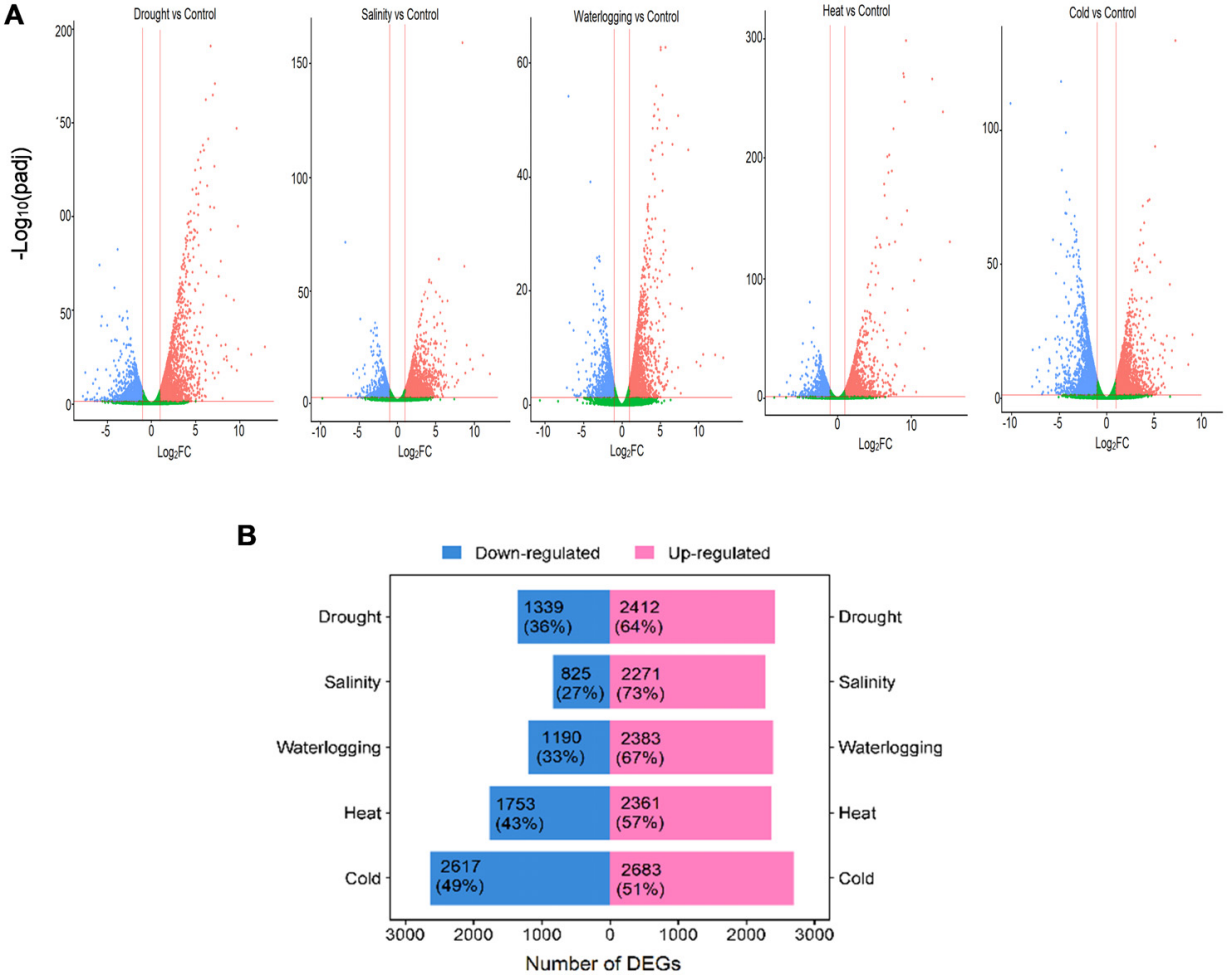
Overview of DEGs in response to abiotic stresses. **(A)** Volcano plots show DEGs of each abiotic stress. Significantly upregulated DEGs (log_2_FC > 1, adjusted *p*-value < 0.05) are highlighted in red dots, and significantly downregulated DEGs (log_2_FC < −1, adjusted *p*-value < 0.05) are highlighted in blue dots. Non-significant genes are shown in green. **(B)** Bar chart shows the total and percentage of upregulated (pink bar) and downregulated (blue bar) DEGs in each abiotic stress.

A comparison between treated and control oil palm samples revealed a total of 19,834 DEGs in response to five abiotic stresses; 12,110 (61%) upregulated and 7,724 (39%) downregulated (**Figures 1A, B**). These DEGs were mainly upregulated under various abiotic stress treatments except cold treatment. The cold treatment resulted in the highest DEGs (5,300) with 2,683 (51%) upregulated DEGs and 2,617 (49%) downregulated DEGs, indicating that these expressed genes were most impacted by cold stress. Oil palm was moderately impacted by heat treatment resulting in 4,114 DEGs, with 2,361 (57%) upregulated and 1,753 (43%) downregulated genes. Drought treatment exhibited a total of 3,751 DEGs; among them, 2,412 (64%) were upregulated and 1,339 DEGs (36%) were downregulated while waterlogging treatment exhibited 3,573 DEGs with 2,383 (67%) upregulated and 1,190 (33%) downregulated genes (**Figure 1B**). Salinity treatment has the least impact on oil palm with the least DEGs (3,096) identified; 2,271 (73%) DEGs were upregulated and 825 (27%) were downregulated (**Figure 1B**).

### 3.2 Functional enrichment classification of DEGs

**Figure 2:**
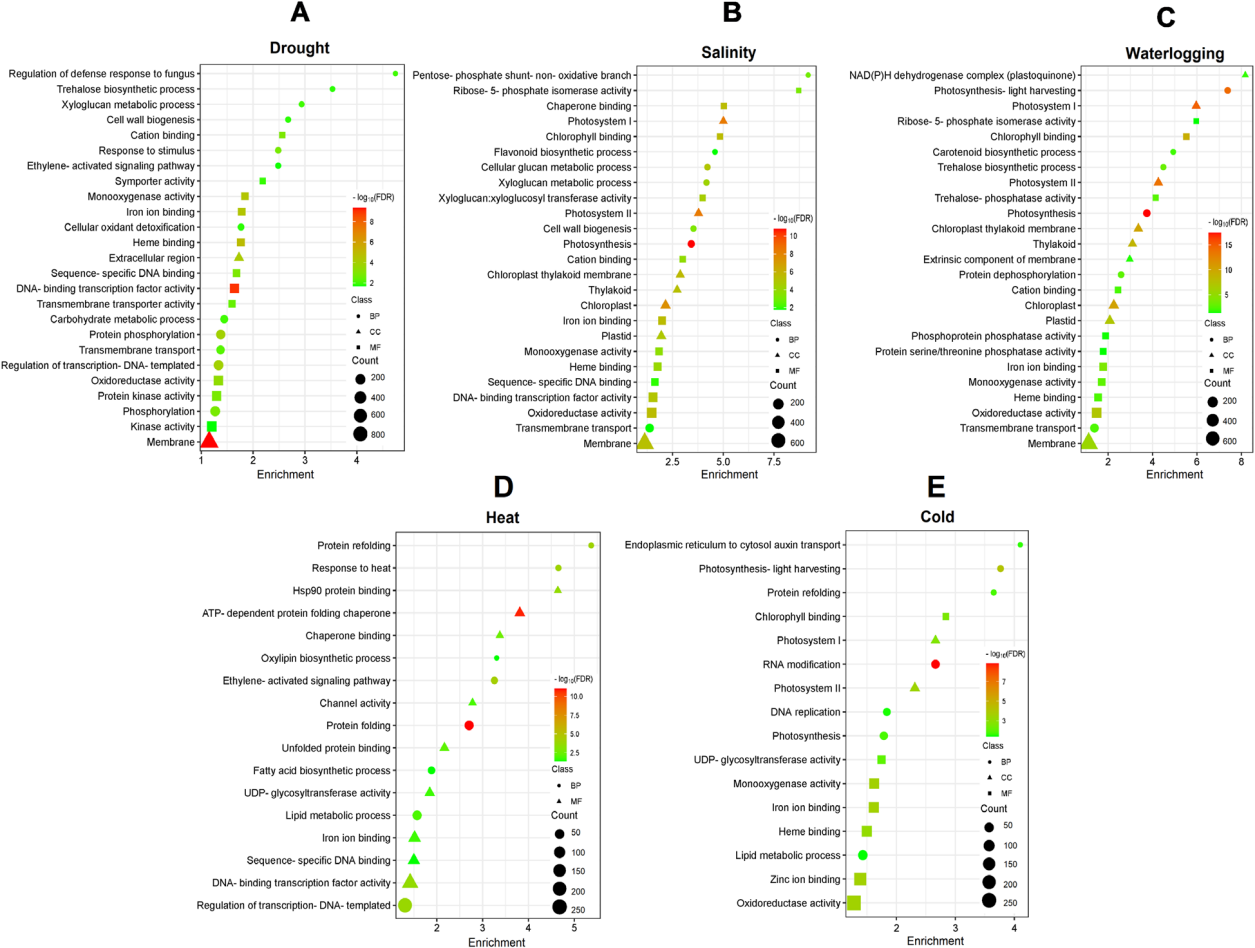
GO enrichment analysis of DEGs under **(A)** drought, **(B)** salinity, **(C)** waterlogging, **(D)** heat, and **(E)** cold treatments. The color gradient from red to green represents the log_10_(FDR) of the enrichment, with red indicating higher significance. The size of each point indicates the count of DEGs associated with the corresponding GO term. Different shapes represent the GO categories: circles for biological process (BP), triangles for cellular component (CC), and squares for molecular function (MF).

To understand the function of these DEGs in response to various abiotic stresses, the GO database was used to perform significant enrichment GO analysis based on biological processes, molecular functions, and cellular components (**Supplementary Table S2**). The most significant (FDR < 0.05) and frequently assigned GO for drought stress include “regulation of defense response to fungus” (GO:1900150), “trehalose biosynthetic process” (GO:0005992), “DNA-binding transcription factor activity” (GO:0003700), and “integral component of membrane” (GO:0016021) (**Figure 2A**). DEGs for both salinity and waterlogging stresses exhibited similar gene enrichment patterns, with the most significant enriched GO involved in cell wall/membrane synthesis and photosynthesis, such as “xyloglucan metabolic process” (GO:0010411), “cell wall biogenesis” (GO:0042546), “photosystem II” (GO:0009523), “chloroplast thylakoid membrane” (GO:0009535), and “chlorophyll binding” (GO:0016168) (**Figures 2B, C**). Meanwhile, the GO terms enriched by DEGs of heat stress were mainly involved in protein protection, fatty acid biosynthesis, and chaperon synthesis, including “protein folding” (GO:0006457), “protein refolding” (GO:0042026), “fatty acid biosynthetic process” (GO:0006633), and “ATP-dependent protein folding chaperone” (GO:0140662) (**Figure 2D**). In cold-treated samples, GO enrichment analyses revealed that “photosynthesis-light harvesting” (GO:0009765) and “protein refolding” (GO:0042026) were the most enriched biological process, “photosystem II” (GO:0009523) was the most enriched cellular component, and “iron ion binding” (GO:0005506) and “oxidoreductase activity” (GO:0016491) were the most enriched molecular function (**Figure 2E**).

### 3.3 Pathway enrichment analysis of DEGs

**Figure 3:**
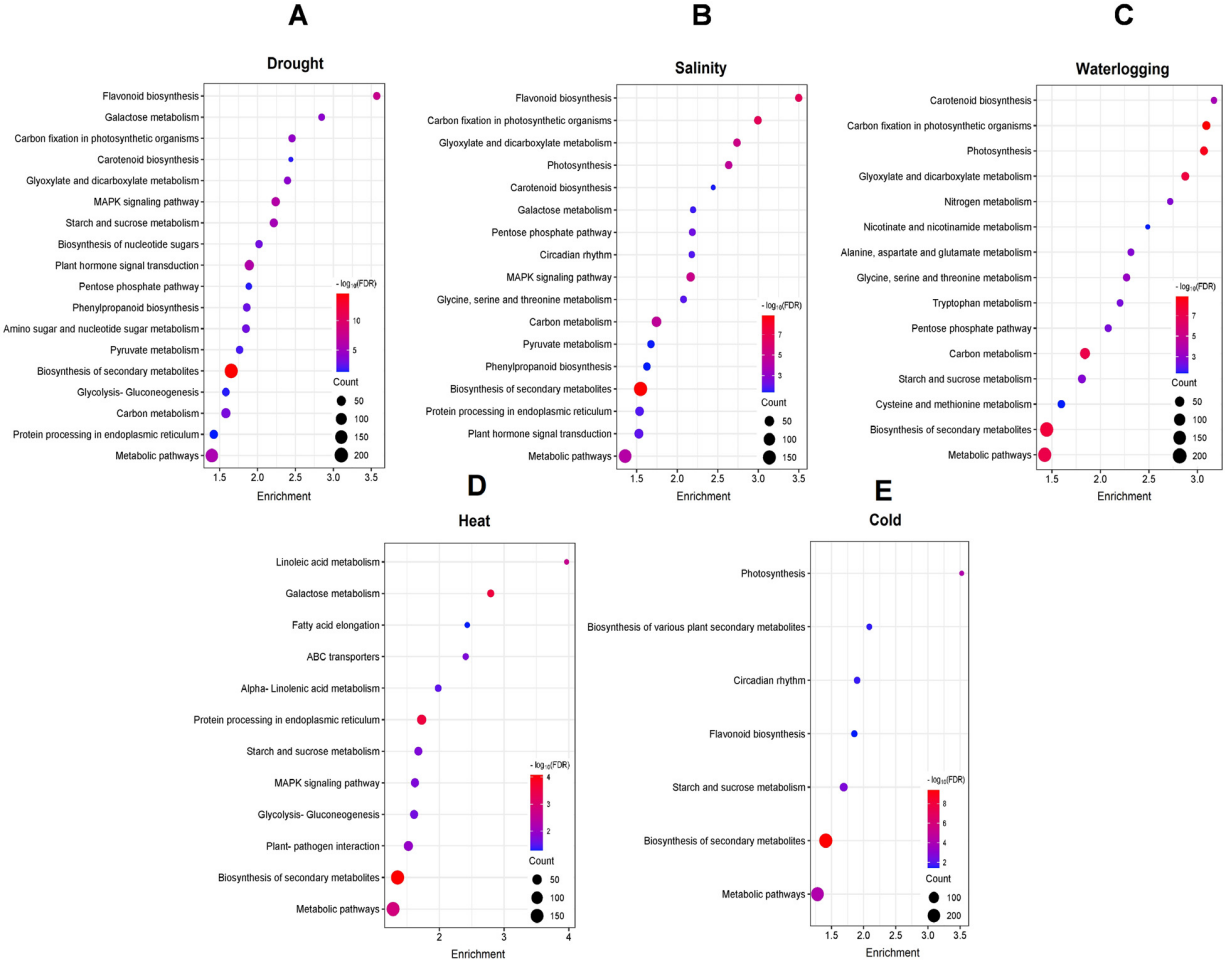
KEGG pathway enrichment analysis of DEGs under **(A)** drought, **(B)** salinity, **(C)** waterlogging, **(D)** heat, and **(E)** cold treatments. The color gradient from red to blue represents the log_10_(FDR) of the enrichment, with red indicating higher significance. The size of each point indicates the count of DEGs associated with the corresponding pathway.

We conducted KEGG enrichment analysis of DEGs to identify enriched metabolic pathways involved in various abiotic stresses. Those DEGs from drought- and salinity-treated samples were enriched in 18 pathways, while 16 enriched pathways were identified in waterlogging-induced DEGs. Heat-induced DEGs were enriched in 12 pathways, and cold-induced DEGs were enriched in only 7 pathways (**Figures 3**). The KEGG analysis showed that DEGs for drought stress were most significantly enriched in pathways including “flavonoid biosynthesis” (egu00941), “galactose metabolism” (egu00052), and “carbon fixation in photosynthetic organisms” (egu00710) (**Figure 3A**). For salinity stress, DEGs were most significantly enriched in “flavonoid biosynthesis” (egu00941), “carbon fixation in photosynthetic organisms” (egu00710), and “glyoxylate and dicarboxylate metabolism” (egu00630) pathways (**Figure 3B**). In waterlogging, those DEGs were mostly enriched in pathways involving “carotenoid biosynthesis” (egu00906), “carbon fixation in photosynthetic organisms” (egu00710), and “photosynthesis” (egu00195) (**Figure 3C**), while the heat-induced DEGs were significantly and specifically enriched in fatty acid biosynthesis related pathways, such as “linoleic acid metabolism” (egu00591), “galactose metabolism” (egu00052), and “fatty acid elongation” (egu00062) (**Figure 3D**). Moreover, the most enriched pathways for cold induced DEGs were “photosynthesis” (egu00196), “biosynthesis of various plant secondary metabolites” (egu00999), and “circadian rhythm” (egu04712) (**Figure 3E**).

Notably, several pathways were unique and specifically enriched by DEGs associated with a particular abiotic stress. Drought-induced DEGs were enriched uniquely in the “amino sugar and nucleotide sugar metabolism” (egu00520) pathway. Pathways associated with amino acid biosynthesis, including “alanine, aspartate and glutamate metabolism” (egu00250), “cysteine and methionine metabolism” (egu00270), and “tryptophan metabolism” (egu00380) were enriched specifically in the waterlogging-induced DEGs. Under heat stress, DEGs were enriched uniquely in pathways like “ABC transporters” (egu02010) and “plant-pathogen interaction” (egu04626) while cold-induced DEGs were enriched uniquely in the “circadian rhythm” (egu04712) pathway. These results suggest the involvement of two distinct regulatory networks including a crosstalk mechanism between various abiotic stress responses and the unique mechanism for each abiotic stress response in oil palm.

### 3.4 DEGs commonly expressed in five abiotic stresses constitute oil palm core abiotic stress transcriptome

**Figure 4:**
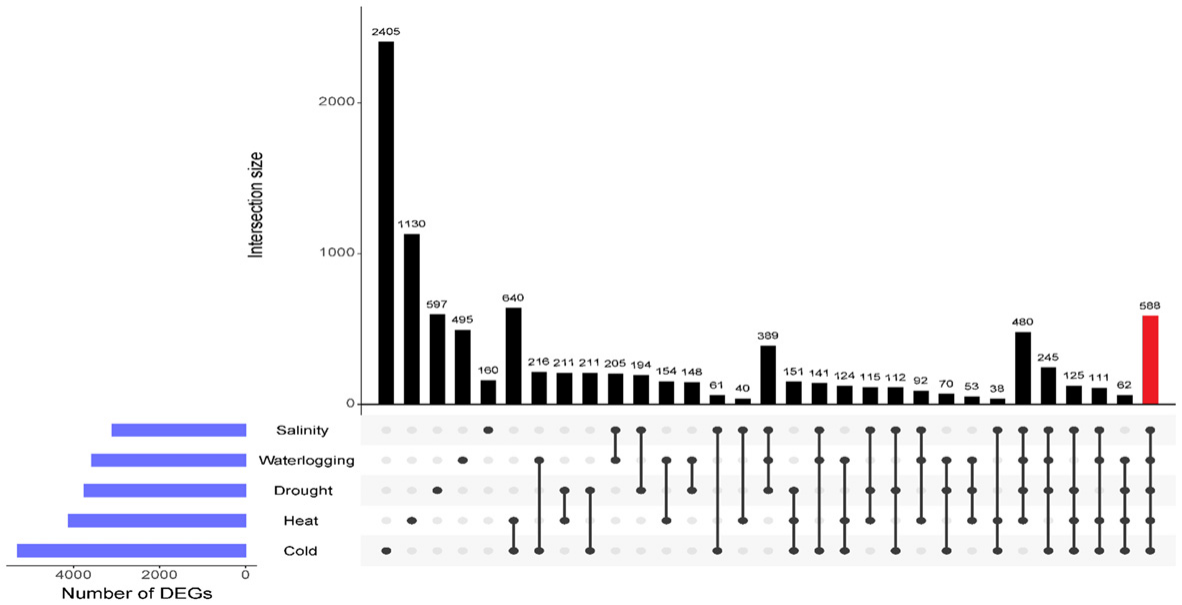
UpSet plot shows the intersection of DEGs across different comparisons of abiotic stress-treated samples. The horizontal blue bars on the left represent the total number of DEGs for each abiotic stress. The vertical bars represent the size of the intersections between gene sets of different comparisons as shown by the individual dots and connecting lines. DEGs common to five abiotic stresses are highlighted in red bar.

We further identified those genes uniquely expressed within the stress treatment or co-expressed by multiple stresses using UpSet plot analysis. An enhanced number of unique DEGs were identified under cold and heat treatments, with 2,405 DEGs and 1,130 DEGs, respectively, indicating a greater sensitivity of oil palm towards temperature variations (**Figure 4**). A total of 597 DEGs were expressed specifically in drought stressed samples, 495 DEGs were unique to waterlogging treatment, and 160 unique DEGs were induced by salinity stress. **Table 2** shows the 10 prominently upregulated DEGs unique to the specific abiotic stress treatment. Moreover, 588 core DEGs were identified as commonly expressed genes in drought, salinity, waterlogging, heat, and cold stresses (**Supplementary Table S1F**).

**Table 2:** The 10 prominently upregulated DEGs that are unique to the abiotic stress treatment only.

Abiotic stress	Gene ID	log_2_ FC	Description
Drought	LOC105046845	5.710	Glycine-rich cell wall structural protein 1
	LOC105058700	4.516	Protein MOTHER of FT
	LOC105035248	4.380	Uncharacterized LOC105035248
	LOC105056444	4.370	Anthocyanin regulatory C1 protein-like
	LOC105041816	4.233	Aluminum-activated malate transporter 9-like
	LOC105035682	4.099	Wall-associated receptor kinase 2-like
	LOC105045265	3.809	Probable esterase PIR7A
	LOC105035099	3.441	Uncharacterized LOC105035099
	LOC105039877	3.428	Ethylene-responsive transcription factor
	LOC105043480	3.418	Uncharacterized LOC105043480
Salinity	LOC105044940	5.193	Peroxidase 5-like
	LOC105053746	4.525	Serine/arginine repetitive matrix protein 2-like
	LOC105033318	4.020	Peroxidase 5-like
	LOC105048187	3.989	Cysteine proteinase inhibitor 1-like
	LOC105039791	3.904	Uncharacterized LOC105039791
	LOC105043536	3.863	1-Aminocyclopropane-1-carboxylate synthase
	LOC105035166	3.858	Uncharacterized LOC105035166
	LOC105061250	3.724	Polygalacturonase inhibitor-like
	LOC105043782	3.277	B3 domain-containing protein REM10-like
	LOC105049227	3.173	Probable xyloglucan endotransglucosylase
	LOC105042452	3.046	Auxin-induced protein 10A5-like
Waterlogging	LOC105032992	8.633	Uncharacterized LOC105032992
	LOC105037078	6.508	Probable LRR receptor-like protein kinase
	LOC105034637	6.320	Pyrophosphate-energized vacuolar membrane proton pump
	LOC105056421	5.569	ORM1-like protein 2
	LOC105045032	5.164	Nicotianamine synthase 3
	LOC105034244	4.948	Uncharacterized LOC105034244
	LOC105047441	4.418	Histidine-containing phosphotransfer protein 4
	LOC105051602	4.406	Lysine histidine transporter 1-like
	LOC105038469	4.312	Amino acid permease 3
	LOC105059530	3.713	Protein TIFY 5A-like
Heat	LOC105048567	8.866	Luminal-binding protein 5-like
	LOC105033962	7.556	Beta-1,3-endoglucanase
	LOC105059654	7.031	Uncharacterized LOC105059654
	LOC105050327	6.936	Aldehyde dehydrogenase 2B7
	LOC105060008	6.555	Flavonol synthase/flavanone 3-hydroxylase
	LOC105032553	6.341	1-Aminocyclopropane-1-carboxylate oxidase
	LOC105036086	6.277	Cytochrome P450 81E8-like
	LOC105038818	5.860	Estradiol 17-beta-dehydrogenase 8-like
	LOC105033416	5.748	Uncharacterized LOC105033416
	LOC105046187	5.641	GDSL esterase
Cold	LOC105032798	5.620	Transcriptional adapter ADA2-like
	LOC105041031	5.426	Uncharacterized LOC105041031
	LOC105037670	5.419	Lysine-specific demethylase JMJ25-like
	LOC105061178	5.345	Phosphoribulokinase
	LOC105035300	5.261	Uncharacterized LOC105035300
	LOC105034790	5.226	Serine/threonine-protein kinase
	LOC105061278	5.051	Uncharacterized LOC105061278
	LOC105042933	4.912	Probable cysteine proteinase A494
	LOC105042611	4.851	E3 ubiquitin-protein ligase ATL23-like
	LOC105046063	4.776	Dihydrolipoyllysine-residue acetyltransferase

**Figure 5:**
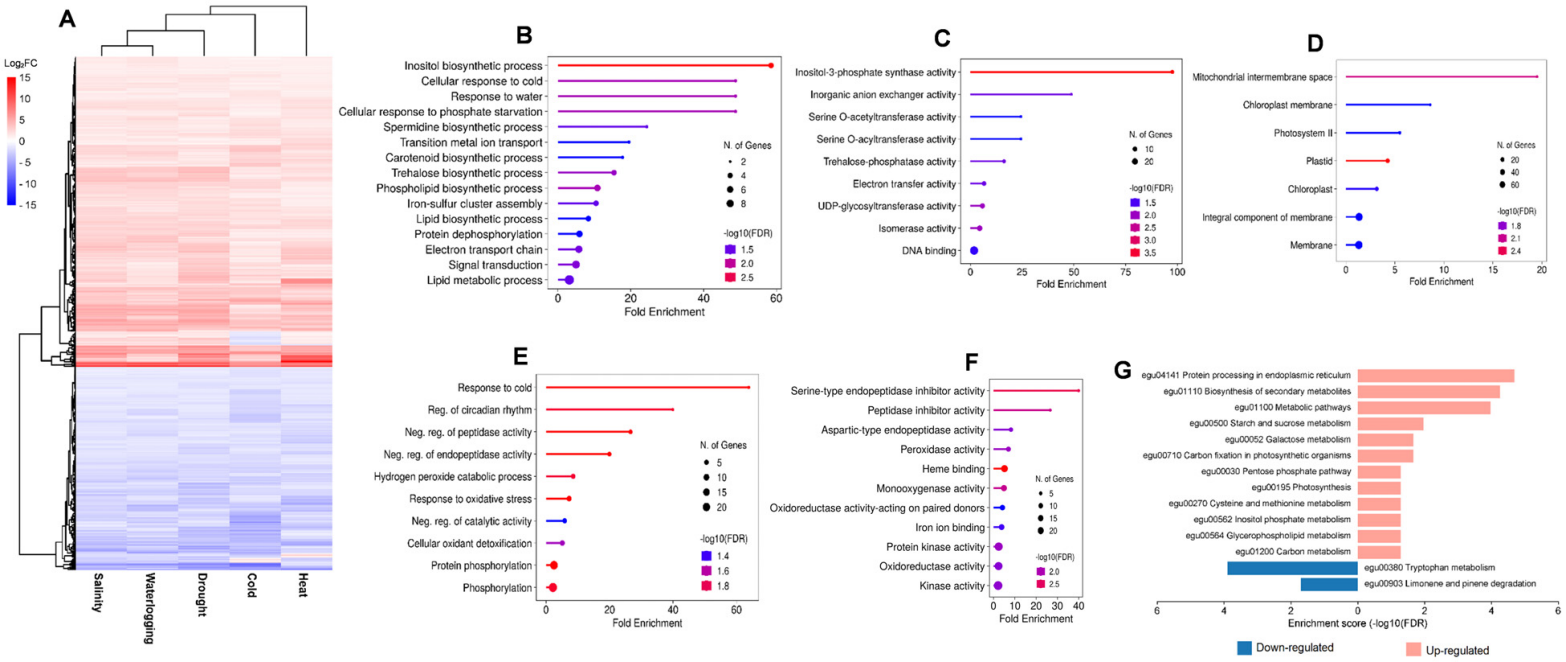
Expression profiles of core DEGs of five abiotic stresses and the enrichment analysis. **(A)** Hierarchical clustering analysis of expression patterns for all core DEGs for salinity, waterlogging, drought, and cold and heat stress that segregated DEGs into upregulated and downregulated cluster. Gene ontology enrichment analysis for upregulated core DEGs in the **(B)** biological process (BP) category, **(C)** molecular function (MF) category, and **(D)** cellular component (CC) category. Gene ontology enrichment analysis for downregulated core DEGs in the **(E)** biological process (BP) category and **(F)** molecular function (MF) category. **(G)** KEGG pathway enrichment analysis of core DEGs in upregulated (pink bar) and downregulated (blue bar) cluster.

**Figure 6:**
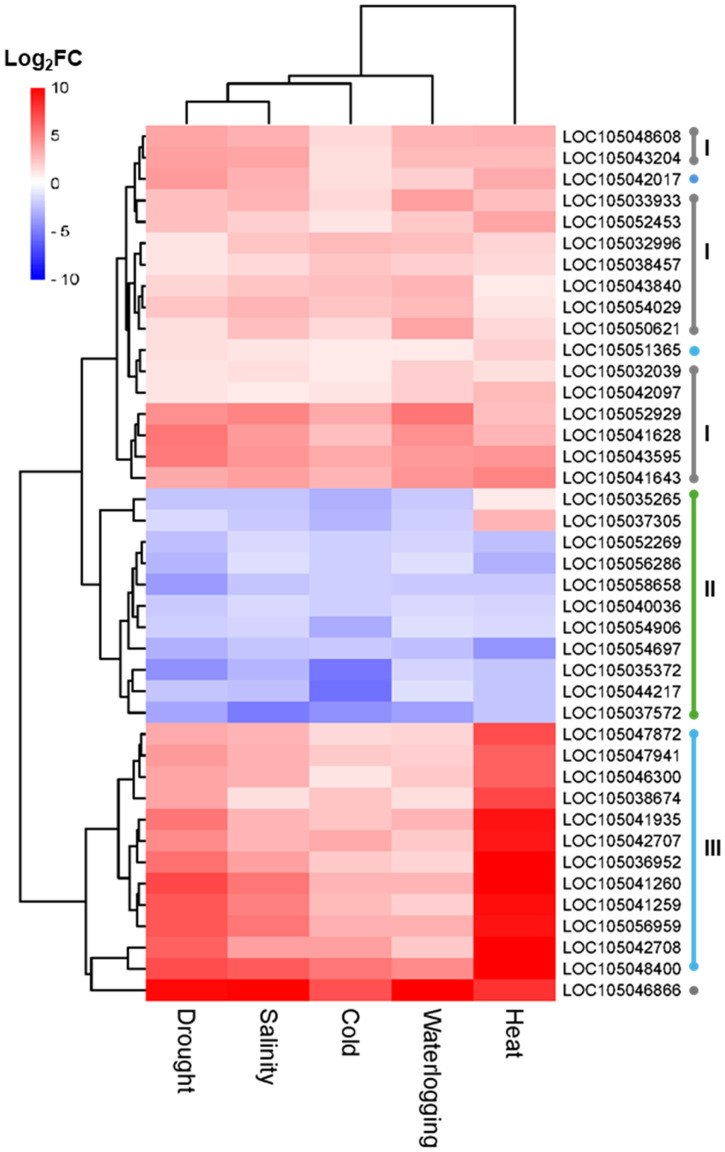
Hierarchical clustering analysis of expression patterns of core DEGs identified in drought, salinity, cold, waterlogging, and heat samples that significantly enriched in the biosynthesis of secondary metabolites (egu01110, gray dot, I), heme binding (GO:0020037, green dot, II), and protein processing in endoplasmic reticulum (egu04141, blue dot, III).

Hierarchical clustering analysis clearly categorized the core 588 DEGs into two main clusters: upregulated cluster (355 DEGs) and downregulated cluster (233 DEGs), despite the fact that several DEGs were expressed inversely in heat and cold stress (**Figure 5A**). The 355 upregulated DEGs were significantly enriched in 18 GO terms in the biological process (**Figure 5B**), 9 GO terms in molecular activity (**Figure 5C**), and 7 GO terms in cellular components (**Figure 5D**). The top enriched GO terms in biological process were “inositol biosynthetic process” (GO:0006021), “cellular response to cold” (GO:0070417), and “response to water” (GO:0009415). In molecular function, the top enriched GO terms were “inositol-3-phosphate synthase activity” (GO:0004512), “inorganic anion exchanger activity” (GO:0005452), and “serine O-acetyltransferase activity” (GO:0009001). In cellular component, GO terms related to photosynthesis such as “photosystem II” (GO:0009523) and “chloroplast membrane” (GO:0031969) were significantly enriched. Meanwhile, the downregulated 233 DEGs were significantly enriched in 10 GO terms in biological process (**Figure 5E**) such as “protein phosphorylation” (GO:0006468) and “response to oxidative stress” (GO:0006979) (**Figure 5F**). Among the 11 GO terms in molecular function, “serine-type endopeptidase inhibitor activity” (GO:0004867) and “heme binding” (GO:0020037) were the most significantly enriched GO. Further heatmap analysis on the GO term “heme binding” showed enrichment of DEGs that were members of cytochrome P450 (**Figure 6**).

The KEGG pathway enrichment analysis showed that “tryptophan metabolism” (egu00380) and “limonene and pinene degradation” (egu00903) were the most significant enriched pathways in the downregulated core DEGs (**Figure 5G**). For upregulated core DEGs, the most significant enriched pathways include “protein processing in endoplasmic reticulum” (egu04141), “biosynthesis of secondary metabolites” (egu01110), and “metabolic pathways” (egu01100) (**Figure 5G**). DEGs enriched in the “protein processing in endoplasmic reticulum” pathway consist primarily of sHSP and HSP, suggesting the importance of sHSP and HSP as key players in the oil palm abiotic stress response mechanism (**Figure 6**). In addition, we identified alpha-terpineol synthase and inositol-3-phosphate synthases from DEGs enriched in the “biosynthesis of secondary metabolites” pathway (**Figure 6**).

### 3.5 Identification of core and unique transcription factors in response to abiotic stresses

**Figure 7:**
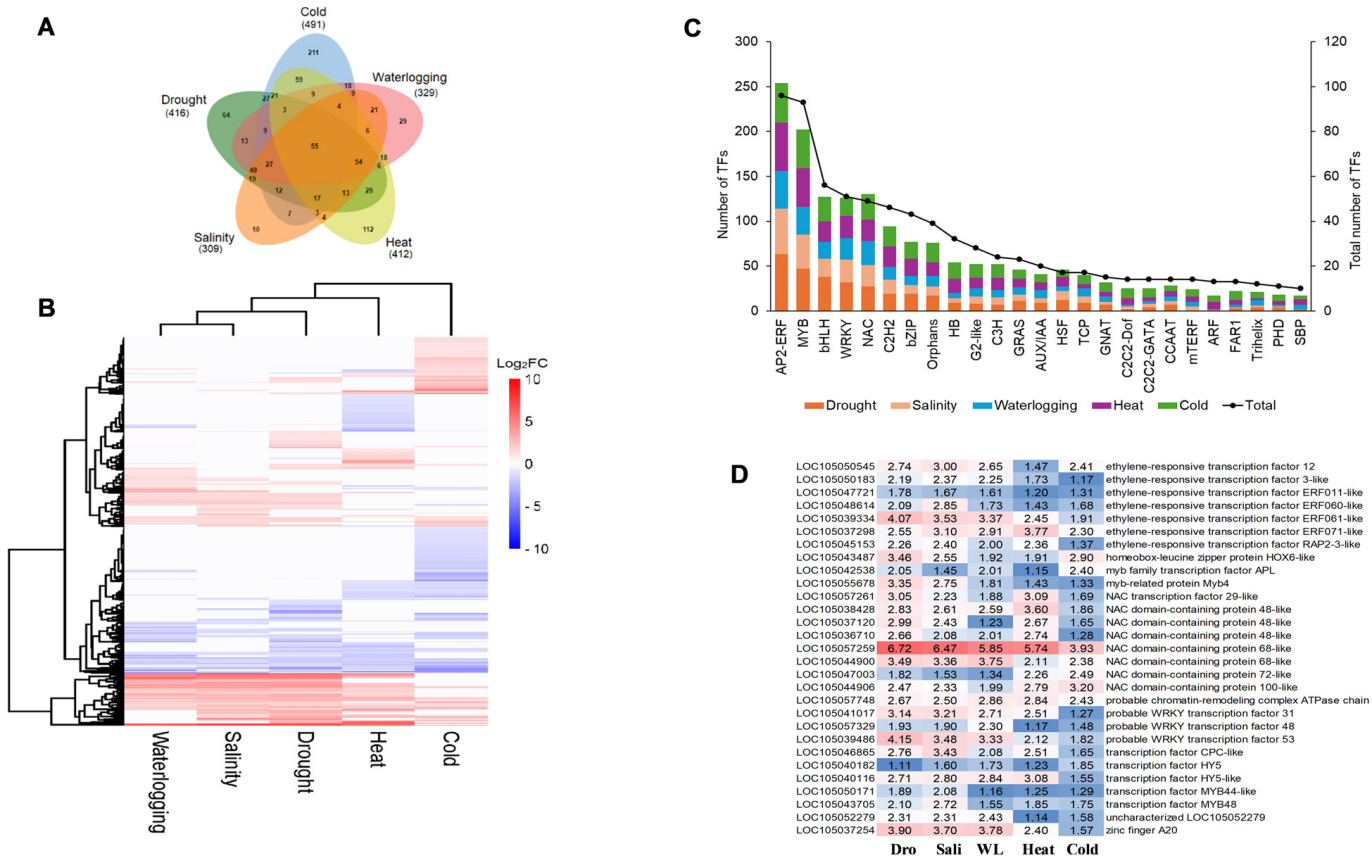
Expression profile analysis of DEGs encode for TF. **(A)** Venn diagram of DEGs encode for TF indicates common and unique TF under different abiotic stress conditions. Numbers in bracket shows total number of DEGs identified. **(B)** Hierarchical clustering analysis of expression patterns for TF encoding DEGs identified in waterlogging, salinity, drought, and heat and cold samples. **(C)** Distribution of the top 25 abiotic stress-related transcription factor families identified in drought, salinity, waterlogging, and heat- and cold-treated samples. Analysis of DEGs encode for TF in response to drought, salinity, waterlogging, and heat and cold stress. **(D)** Heatmap of the 29 core DEGs encode for TF of oil palm abiotic stress response. The color gradient from red to blue represents the fold change (FC) of upregulated DEGs, with red indicating higher FC level. The numbers inside the cells represent the fold change (FC) values. Dro, drought; Sali, salinity; WL, waterlogging.

Transcription factors are central regulators that modulate the expression of abiotic stress-responsive genes in plants. Plant TF database, namely, iTAK, was used to explore TFs among those DEGs induced by five abiotic stresses. A total of 936 TFs were identified from those DEGs induced by five abiotic stresses and categorized into more than 50 different TF families. Cold stress induced the highest number of TFs at 491, followed by drought stress (416 TFs), heat stress (412 TFs), waterlogging stress (329 TFs), and salinity stress (309 TFs) (**Figure 7A**). Among these TFs, 211 TFs were uniquely induced by cold stress, 112 TFs specifically induced by heat stress, 64 TFs unique to drought stress, 29 TFs unique to waterlogging stress, and 10 TFs specific to salinity stress (**Figure 7A**).

Hierarchical clustering of TFs encoding DEGs revealed the complexity of transcriptional regulatory networks and the dynamic nature of gene expression in response to various abiotic stresses (**Figure 6B**). Among these 50 TF families, the most enriched TF families included ethylene-responsive transcription factor (AP2-ERF); myeloblastosis (MYB) TFs; basic helix-loop-helix TFs (bHLH); WRKY TFs; NAM, ATAF1/2, and CUC2 (NAC) TFs; zinc finger TFs (C2H2); basic leucine zipper TFs (bZIP); heat stress TFs (HSF); and auxin response factor (ARF) (**Figure 7C**). Moreover, we identified 55 TFs encoding DEGs that are commonly expressed in five abiotic stresses, constituting the core TFs in oil palm abiotic stress response (**Figure 7A**). The heatmap of the 29 core TFs encoding DEGs that were significantly enriched in the GO term “DNA binding” (GO:0003677) revealed the potential of NAC TFs and AP2-ERF TFs as universal regulators in modulating oil palm abiotic stress response (**Figure 7D**).

### 3.6 Validation of RNA-Seq data using qRT-PCR

**Figure 8:**
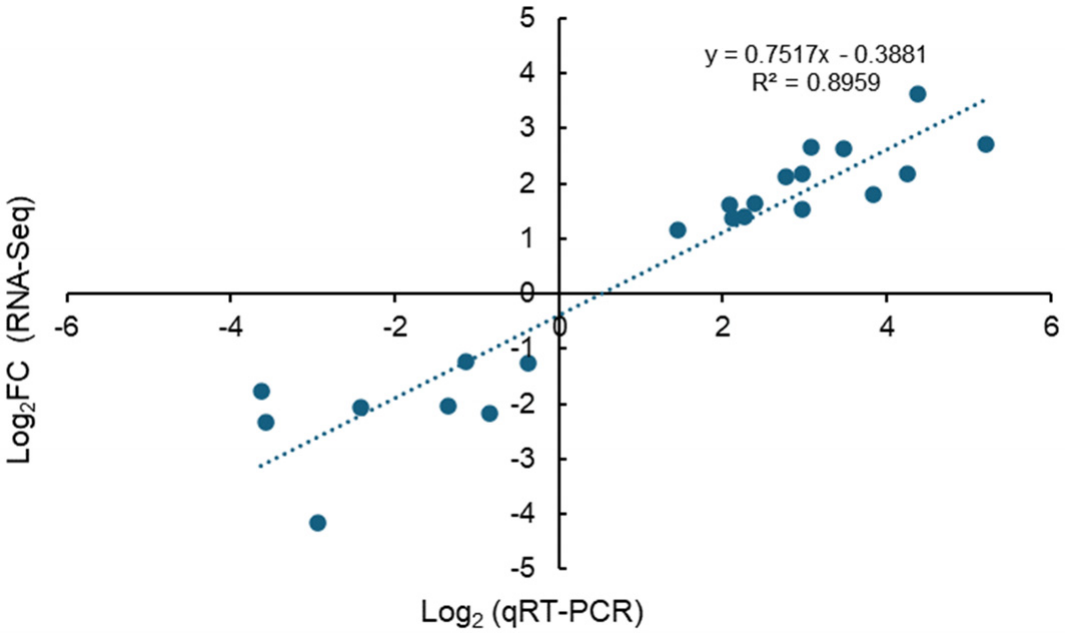
qRT-PCR validation of DEGs characterized by RNA-Seq. Correlation of fold change identified by the RNA-Seq method (*x*-axis) with expression data obtained using the qRT-PCR method (*y*-axis) to validate DEGs characterized by RNA-Seq.

To validate the reliability of high-throughput transcriptome sequencing, 16 DEGs were randomly selected for gene expression qRT-PCR analysis. The correlation of targeted DEG expression levels was identified by comparing the relative quantities from qRT-PCR against the fold change from RNA-Seq analysis. Real-time PCR and RNA-Seq have a high correlation with *r* ^2^ = 0.8959 (*n* = 22), thus suggesting the high confidence level of the reliability of expression levels of DEGs identified from RNA-Seq data (**Figure 8**).

## 4 Discussion

### 4.1 Pronounced transcriptomic changes observed under temperature variations

Oil palm is constantly exposed to various abiotic stresses, resulting in poor yield performance, especially if the unfavorable conditions worsen and persist (Abubakar et al., 2022). We conducted transcriptomic studies to elucidate the DEGs involved in oil palm responses to different abiotic stresses. An MOI study of oil palm in response to drought and salinity revealed L-serine O-acetyltransferase and cysteine synthase as two key enzymes involved in the cysteine and methionine metabolisms, which were upregulated under both stresses (Leão et al., 2022). The root transcriptomic responses of oil palm seedlings under 14-day drought stress revealed that 1,293 DEGs involved in cell wall biogenesis and functions, phenylpropanoid biosynthesis and metabolisms, and ion transport and homeostasis were significantly enriched in hormone regulation and metabolism and ABC transporters pathways (Wang et al., 2020). In response to waterlogging stress, the hypoxia-related TF HRE2, which belongs to the ERF-VII TF family, was postulated to play an important role in adaptation to hypoxia and ethylene signaling in the adult oil palm stems (Lim et al., 2023). These findings establish a foundation for oil palm responses to individual or dual abiotic stresses.

Here, we compared 18 transcriptomic libraries to gain a deeper understanding of oil palm responses to various abiotic stresses. Our transcriptome analysis revealed that oil palm reprograms its transcriptome profile to modulate the gene expression of both unique and common networks in response to single or multiple abiotic stresses. We observed that oil palm seedlings exhibited greater sensitivity to temperature variations compared to the osmotic stress caused by drought, salinity, and waterlogging. A higher number of DEGs were induced by cold (5,300) and heat (4,114) stresses as compared to drought (3,751), salinity (3,573), and waterlogging (3,096) stresses (**Figure 1**). Cold stress-induced DEGs mainly involved in auxin-regulated responses and enriched in GO “endoplasmic reticulum to cytosol auxin transport” and “monooxygenase activity” in oil palm are also observed in the oil palm leaf transcriptome subjected to cold treatment (Saand et al., 2022). Conversely, the increase in temperature due to heat stress treatment in oil palm significantly altered the expression of genes related to protein protection, fatty acid biosynthesis, and chaperone synthesis, suggesting that these metabolic changes protect oil palm from irreversible cell membrane degradation and protein denaturation, ensuring their survival under abiotic stress. Rapid transcriptional changes have been reported in *Brassica*, *Arabidopsis*, and legume in response to variations in temperature, to maintain protein stability, membrane fluidity, cellular integrity, and developmental processes through hormonal regulation and auxin (Shibasaki et al., 2009; Mehrotra et al., 2020; Sohrabi et al., 2022). Under cold stress, auxin transport is regulated to inhibit the intracellular trafficking of auxin efflux carrier proteins, leading to reduction in gravity response and prevents elongation in plant roots (Shibasaki et al., 2009). This serves as a stress avoidance or protective mechanism that prevents cold stress impact on plants. Furthermore, a lower degree of transcriptional changes was observed in oil palm seedlings under drought, salinity, and waterlogging stresses. Gradual and specific long-term adaptive mechanisms are activated to regulate water and ion transport, osmo-protectant synthesis, and TF regulation from the GO and pathway enrichment analyses. These results suggest that oil palm stress response is dynamic and involves extensive gene expression adjustment for cellular and physiological adaptation, particularly in response to temperature variations compared to the short-term osmotic stress.

### 4.2 Core abiotic stress transcriptome of oil palm

**Figure 9:**
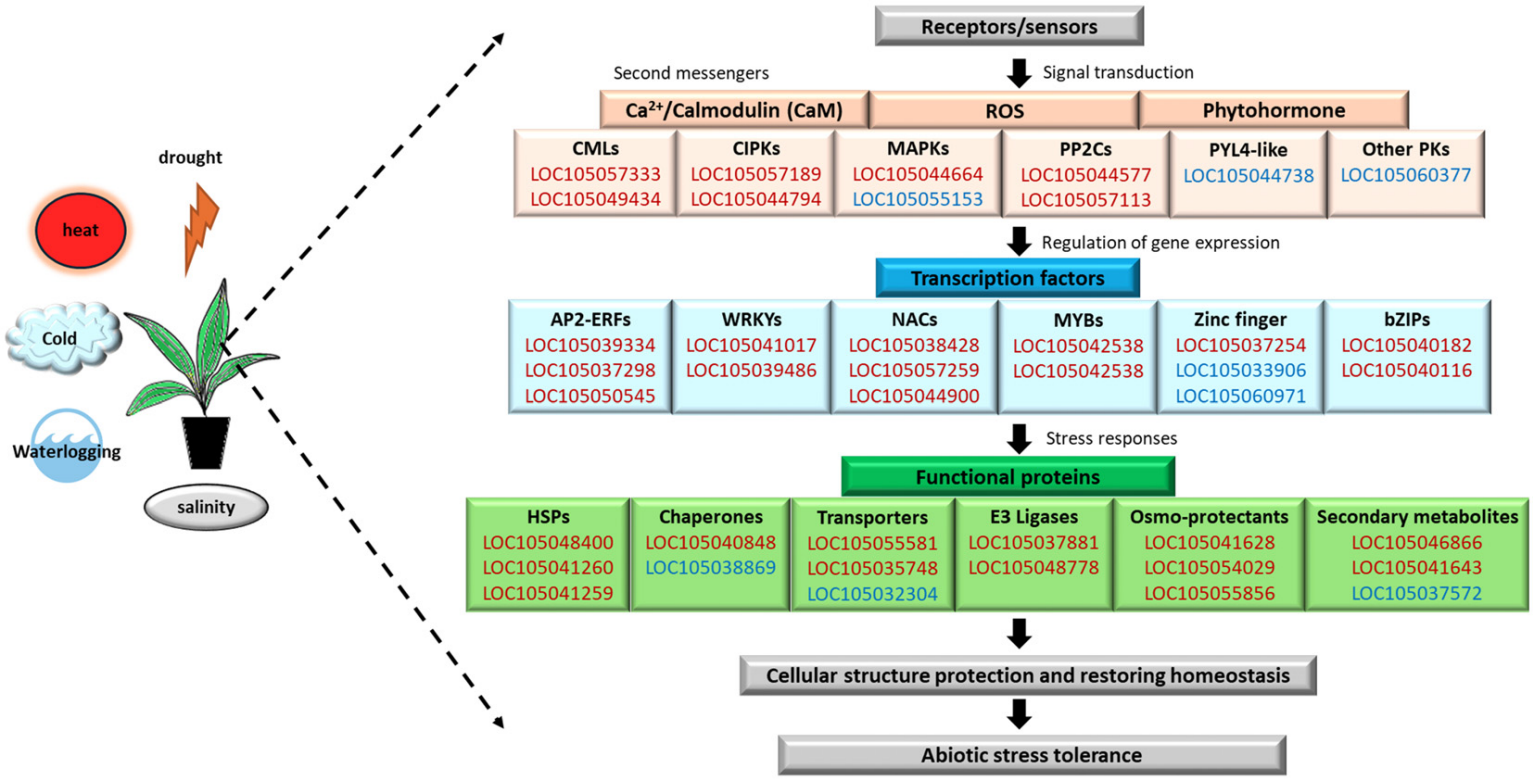
Overview of the CAST of oil palm abiotic stress signaling pathways in oil palm highlighting the core upregulated DEGs (red) and downregulated DEGs (blue), which are involved in the signal transduction, gene regulation, stress responses, and abiotic stress tolerance.

The ability of the plant to perceive external environmental cues and transduce precise signal rapidly is critical to trigger molecular, cellular, physiological, biochemical, and morphological changes, to maintain optimal plant growth and development under adverse conditions. Plants have evolved sophisticated mechanisms involving complex crosstalk between multiple metabolism, regulatory, and signaling networks under a core response system to achieve cellular homeostasis. Previous studies have reported that the abiotic stress responses in *Arabidopsis* (Hahn et al., 2013), *Sesamum* (Dossa et al., 2019), and *Brassica* (Zhang et al., 2019) led to the identification of core abiotic stress-responsive genes, regulators, functional proteins, and metabolites. Here, our comparative transcriptome analyses revealed the CAST of oil palm. The oil palm CAST comprising 588 DEGs consistently expressed across all five abiotic stresses with 355 upregulated DEGs and 233 downregulated DEGs. Both GO and pathway enrichment analyses showed involvement of CAST in signal transduction, gene expression regulation, and stress response mechanisms (**Figure 9**).

Protein kinases are central components that regulate the activity of critical functional proteins through phosphorylation, in response to environmental stress for adaptation in plants. We identified core DEGs encoding protein kinases in signaling pathway activated by secondary messengers, such as Ca^2+^, ROS, and phytohormones in oil palm (**Figure 9**). Different Ca^2+^ sensors were found upregulated in response to multiple abiotic stresses, including two DEGs (LOC105057333 and LOC105049434) encoding CaM-like proteins (CMLs) and two DEGs (LOC105057189 and LOC105044794) encoding for CBL-interacting protein kinases (CIPKs) (**Supplementary Table S1**). Several DEGs encoding mitogen-activated protein kinase (MAPK) cascade (LOC105044664 and LOC105055153) and protein phosphatases 2C (PP2Cs; LOC105044947, LOC105044577, and LOC105057113) and numerous DEGs encoding for serine/threonine-specific protein phosphatases (PPPs, 14 DEGs) were involved in the ABA signaling pathway (**Figure 9**). Furthermore, phytohormone-related DEGs such as an auxin-repressed 12.5-kDa protein (LOC105042011 and LOC105053050) and ABA receptor PYL4-like (LOC105044738) that showed downregulation in response to multiple abiotic stresses were also identified in the oil palm CAST. These core DEGs are also identified as components of the CAST in rice (Cohen and Leach, 2019) and sesame (Dossa et al., 2019). In rice, overexpression of CIPKs enhanced transgenic rice tolerance to cold and drought stress with increased accumulation of proline and soluble sugar than wild type (Xiang et al., 2007). Likewise, MAPK-based signaling enhanced stress-induced proline accumulation exhibited in *Arabidopsis* overexpressing a maize MAPK gene, under cold and salt stress treatments (Kong et al., 2011). Proline acts as an osmolyte, ROS scavenger, and chaperone that stabilizes proteins and protects cells in response to various abiotic stresses; hence, this suggests the correlation between protein kinases and signaling in proline metabolism for stress response. Moreover, three upregulated core DEGs (LOC105048608, LOC105032996, and LOC105041643) significantly enriched in the GO term “inositol-3-phosphate synthase activity” (GO:0004512) were identified in the oil palm CAST. Inositol-3-phosphate synthase is involved in the hydrogen sulfide (H_2_S) regulation of salt stress tolerance in cucumber (Jiang et al., 2020) and the crosstalk of Ca^2+^, phytohormone, and ROS signaling pathways (Jia et al., 2019). Inositol-3-phosphate synthase of oil palm may play a vital role in orchestrating the signal transduction of external stimuli involving core DEGs encoding kinases. This result suggests that oil palm CAST DEGs may play an important role in signaling transduction to allow rapid and concise abiotic stress responses under the regulation of core TFs, proteins, and metabolism.

Plants develop various strategies to protect and maintain the functionality of proteins during the onset of abiotic stress to ensure a higher survival. We identified core DEGs associated with the ubiquitin–proteasome pathway and protein stability in the oil palm CAST (**Figure 9**). The *E3 ubiquitin-protein ligase BOI-like* and *desumoylating isopeptidase 1* were upregulated in response to multiple abiotic stresses, suggesting their roles in protein modification and turnover under stress conditions. Conversely, DEGs such as the *U-box domain-containing protein 33-like*, *RING-H2 finger proteins*, and *Bowman-Birk type trypsin inhibitor-like* were downregulated, suggesting a fine-tuned balance in protein regulation. A study in rice reported that the *RING finger protein 1* (*OsDHSRP1*) negatively regulates abiotic stress-responsive gene expression under drought, heat, and salt stress conditions (Kim et al., 2020). Overexpression of the *Small Ubiquitin-Like Modifier protease OTS1* gene enhances drought tolerance in sugarcane, and this further emphasizes the role of ubiquitin-related pathways in stress resilience (Masoabi et al., 2023). Additionally, an ion toxicity abiotic stress study in oil palm revealed that the gene encoding Bowman-Birk-type trypsin inhibitor-like, a member of the protease inhibitor family involved in biological-defensive functions, exhibited the highest score in co-expression networks under aluminum stress response (Mejia-Alvarado et al., 2023). These results further provide insight into oil palm’s diverse molecular strategies for progressive responsive adjustment to maintain protein stability and function during early exposure to various abiotic stresses.

Furthermore, we discovered downregulation of six core DEGs encoding for cytochrome P450 (CYP) enzymes across five abiotic stresses in oil palm. CYPs are multifunctional oxidoreductase enzymes that contribute significantly to plant stress response, growth, and development processes by controlling the levels of hormones, fatty acids, sterols, cell wall components, and secondary metabolites (Chakraborty et al., 2023). The downregulation of CYP genes under stress conditions might be an adaptive strategy that helps plants to conserve energy for other vital metabolic processes for plant adaptation or to enhance the production of terpene compounds instead of terpenoid compounds, which are predominantly involved in plant–insect interaction (Boncan et al., 2020). Terpenes are secondary metabolites that function as sensing molecules or chemical mediators of interactions between plants and the environment. Our oil palm CAST showed upregulation of 3 core DEGs encoding for alpha-terpineol synthase-like (TPS). These TPS genes are crucial for terpenes biosynthesis and have been implicated in abiotic stress response and tolerance in plants, such as *C. sinensis* (Zhou et al., 2020), *R. communis* (Silva de Oliveira et al., 2022), and *G. pentaphyllum* (Ling et al., 2023). Hence, TSP and CYP in the CAST of oil palm may interplay in the strategic shifting of metabolic priorities to promote the synthesis of secondary metabolites like terpenes, to enhance stress tolerance and conserve resources for crucial adaptive functions. Overall, oil palm exhibited stress avoidance strategy that involves a variety of protective mechanisms including activation of stress-responsive and functional proteins, securing protein stability and diverting energy for critical metabolisms, to delay the impact of stress components under the short-term abiotic stress exposure.

### 4.3 Transcription factors as universal regulators in modulating oil palm abiotic stress responses

Plants respond and adapt to various environmental conditions by altering the transcription of various stress-responsive genes. This process is regulated by TFs through complex regulatory networks. In this study, we identified more than 50 different TF families responsive to five abiotic stresses. Among these, the most enriched TF families included AP2-ERF, myeloblastosis (MYB), bHLH, WRKY, NAC, C2H2, basic leucine zipper (bZIP), heat stress TF (HSF), and auxin response factor (ARF). A similar observation was reported in the leaf transcriptome of three oil palm varieties (*Bamenda* × *Ekona*, *Tanzania* × *Ekona*, and *E. oleifera* × *E. guineensis*), whereby MYB, AP2-EREBP, NAC, and WRKY were the most enriched TF families in response to cold stress (Saand et al., 2022). Notably, 55 DEGs were identified as core TFs across five abiotic stresses. Heatmap analysis of the 29 DEGs encoding core TFs revealed the potential of different members of AP2-ERF, MYB, WRKY, and NAC TF as universal regulators in modulating plant responses to multiple abiotic stresses (**Figure 7D**). This finding aligns with previous studies highlighting the significant roles of specific TFs in abiotic stress responses. For instance, AP2-ERF, bZIP, and MYBR1 families of rapeseed were identified as core abiotic stress TFs (Zhang et al., 2019), further highlighting the significance of these TF families in abiotic stress responses. In sesame, 18 TF families including AP2-ERF, MYB, bHLH, and WRKY were identified as core abiotic stress TFs (Dossa et al., 2019). Further investigation of two sesame hub genes, *SiERF5* and *SiNAC104*, in transgenic Arabidopsis exhibited enhanced fitness and performance under abiotic stresses conditions compared to wild type, leading to enhanced tolerance to drought, waterlogging, and osmotic stresses (Dossa et al., 2019). A genome-wide analysis of oil palm stress response also induced TFs including NAC (Xiao et al., 2018), AP2-ERF (Zhou and Yarra, 2021), and bZIP (Zhou and Yarra, 2022), confirming the involvement of these TF families in abiotic stress responses. In addition, overexpression of oil palm *EgMYB111* and *EgMYB157* genes in Arabidopsis enhanced antioxidant enzyme activities and photosynthetic rate, leading to improved tolerance towards salinity, cold, and drought stress (Zhou et al., 2022).

HSP TFs are molecular chaperones essential for maintaining vitality and functionality of proteins under stress conditions. In the pathway enrichment analysis of oil palm CAST, 13 upregulated DEGs were significantly enriched in the “protein processing in endoplasmic reticulum” pathway (egu04141). These 13 DEGs encode for sHSP and various classes of HSPs, highlighting their potential as universal functional proteins in abiotic stress responses. Previous studies in oil palm leaves under drought stress reported that upregulation of *HSP70* (Azzeme et al., 2016) and differential expression pattern of *HSPs* was induced in response to cold stress (Saand et al., 2022) and heat stress (Maryanto et al., 2021). Additionally, *CssHsp-08*, *CsHsp40-70*, and *CsHsp70-06* from cucumber were upregulated under various abiotic stresses (Unel et al., 2023). Similarly, several HSPs have been identified in the CAST of sesame under abiotic stress conditions (Dossa et al., 2019). Furthermore, a study showed that ERF1 of *Arabidopsis* positively regulated heat tolerance by binding to the DRE *cis*-elements in the promoter of *HSP* to activate its expression (Cheng et al., 2013). The Arabidopsis ERF95 and ERF97 are also positive regulators of basal heat stress response that act downstream of ethylene signaling component EIN3 and regulate transcription of heat stress-responsive genes including *HSFA2, HSFA7a*, *HSP20-like*, and *HSPs* (Huang et al., 2020). Coincidentally in oil palm, six core DEGs encoded for ERF were upregulated in oil palm CAST, suggesting that these core HSPs may be regulated by the core ERF to establish protein stability and enhance stress resilience in oil palms. These findings collectively demonstrated the crucial roles of these core TF cascades in regulating downstream stress-responsive genes across various abiotic stress in the oil palm (**Figure 9**).

## 5 Summary

The oil palm comparative study identified DEGs induced by cold stress (5,300 DEGs), heat stress (4,114 DEGs), drought stress (3,751 DEGs), waterlogging stress (3,573 DEGs), and salinity stress (3,096 DEGs). Subsequent analysis unveiled the CAST of oil palm comprising 588 DEGs commonly expressed under drought, salinity, waterlogging, heat, and cold stress conditions. Both GO and pathway enrichment analyses of these DEGs in the CAST suggested their roles in signal transduction, transcription regulation, and abiotic stress responses including synthesis of osmolytes, secondary metabolites, and molecular chaperones. Moreover, we identified core DEGs that encoded for kinases, NAC TFs, HSPs, E3 ubiquitin-protein ligase, terpineol synthase, and cytochrome P450. These core DEGs may be the potential key modulators in the CAST of oil palm to restore homeostasis and enhance palm adaptation to various abiotic stresses. Further validation is required to confirm their function in conferring stress tolerance. Our findings unravel the key modulators within the CAST of oil palm, offering valuable insights for gene marker exploration and potential targets for gene editing to develop climate-resilient planting materials that thrive in unpredictable environments.

## Data Availability

The datasets presented in this study can be found in online repositories. The names of the repository/repositories and accession number(s) can be found below: https://www.ncbi.nlm.nih.gov/, PRJNA775831 https://www.ncbi.nlm.nih.gov/geo/, GSE242922.
